# Baby walker injury, disability, and death in a high-income middle eastern country, as reported by siblings

**DOI:** 10.1186/s40621-016-0082-7

**Published:** 2016-07-12

**Authors:** Peter Barss, Michal Grivna, Amna Al-Hanaee, Ayesha Al-Dhahab, Fatima Al-Kaabi, Shamma Al-Muhairi

**Affiliations:** 1 School of Population and Public Health, University of British Columbia, Vancouver, BC Canada; 2 Institute of Public Health, College of Medicine & Health Sciences, United Arab Emirates University, PO Box 17666, Al Ain, United Arab Emirates

**Keywords:** Babywalker, Infant walker, Baby walker, Child injury, Disability, Mortality, Falls, Epidemiology, Prevention

## Abstract

**Background:**

Baby walkers (BWs) are frequent causes of infant injuries. Little research is reported from the Middle East and few population-based studies anywhere.

**Methods:**

Using multistage random sampling in a city of the United Arab Emirates, 4 of 8 female Arab government high schools and 3 final-year classes each from science and arts tracks were selected. Structured self-administered questionnaires assessed prevalence, frequency, severity, and external causes of BW incidents and injuries, and residential hazards.

**Results:**

Response was 100 %, 696 students, 55 % (*n* = 385) Emirati citizens. 87 % (*n* = 605) of families used/had used BWs. Among 646 injuries were 118 ER (emergency) visits, 42 hospitalizations, 11 disabilities, and 3 deaths. Average risk was 1 incident/user, 1 injury/4 users, 1 ER visit/20, 1 hospitalization/55, 1 disability/200, 1 death/1000. Odds ratios for >1:1 floor levels were 2.3 (95 % confidence interval: 1.2, 4.3) for hospitalization, 16.8 (95 % CI: 2.1, 132.5) disability. Incidents included hitting objects 48 % (*n* = 1322), overturning 23 % (*n* = 632), accessing hazardous objects 17 % (*n* = 473), and falling down stairs 11 % (*n* = 300); 1 % (*n* = 32) fell into swimming pools. In 49 % (*n* = 297/605) of user families, ≥1 child had been injured.

**Conclusions:**

Despite causing many injuries including disabilities and fatalities, BWs were used by nearly all families. Governments should consider Canada’s lead in prohibiting importation, sales, and advertising of BWs.

## Background

Baby walkers (BWs) were described in one of the earliest textbooks of paediatrics in Arabic over a millennium ago (Al-Baladi 1980). Prevalence studies in various countries indicate that this product is used by between 20 and 90 % of parents, mainly for children about 5 to 14 months old (Al-Nouri and Al-Isami 2006; Health Canada 2007; Rodgers and Leland 2005; Santos Serrano et al. 1996). Due to the frequency and severity of BW injuries, the Canadian government has banned import, advertising and sale since 2004 under the Hazardous Products Act, and resisted a subsequent industry challenge (Health Canada 2007). Other countries have no interventions or less effective measures such as warning labels, design modification, or public education (American Academy of Pediatrics 2001; Taylor 2002; Shields and Smith 2006). Despite existing voluntary standards in the United States to prevent falls down stairs, such as presence of brakes and minimum width, not all manufacturers are members of the Juvenile Products Manufacturers Association (JPMA) and receive a safety product certification from JPMA (American Academy of Pediatrics 2001). Furthermore, speeds can be high enough to overcome brakes and incidents such as reaching dangerous objects are not affected by the standards (Health Canada 2002; Ridenour 1997).

Since early description of BWs, little has been published in the Middle East. In an earlier home safety survey in the United Arab Emirates (UAE), a rapidly developing oil-rich country, BWs were used by 32 % of families (Al-Saridi et al. 2005). A larger study was therefore developed focusing specifically on BWs. In a first article we reported the knowledge and attitudes of future mothers on perceived safety, reasons for initiating and stopping use, and support for legislation banning BWs (Grivna et al. 2015). This paper assesses the epidemiology of BW injuries, including prevalence, frequency and severity of associated injuries, external causes, and environmental risks at homes. Due to local Arab culture making it difficult to sample and interview at homes, another method was needed. We chose to interview high school girls as future mothers and as siblings of injured babies. This provided a rapid large multistage random sample.

## Methods

### Design, target population, sampling

A cross-sectional survey was conducted during September 2005 in Al Ain, a desert city of population 460,000, which is one of the four largest cities in the UAE. Using multistage random sampling, 4 of 8 government female Arab high schools were selected, then grade-12 classes in each, including 3 from science and 3 from art tracks. All students in selected classes were included, on average 30 each. Previous research in such schools showed a fairly even distribution of income groups among families (Al-Saridi et al. 2005); 43 % of fathers and 26 % of mothers had at least university education. Children of expatriate workers with very low income would not have been included in sampling since such workers were not allowed to bring families. Because the study was in Arab schools, Indian or European families were not included.

Nowadays many mothers work, delegating oversight and care of young children to older siblings and/or housemaids. Hence, siblings are potentially more aware of home injury incidents than their working mothers. Sample size requirements to detect uncommon events such as deaths and disabilities together with challenges of household surveys of women in the Islamic cultural context indicated that a population-based school survey of youth in generally large families was the most feasible study design.

### Data collection instrument

The self-administered questionnaire included 32 structured questions. It was developed in English and translated into Arabic. BW prevalence and injury incidence for each young woman’s family were reported as far back as they could recall, including only persons living in their home; extended families were excluded. The questionnaire included: socio-demographic variables such as age, nationality, number of children in the family <15 years-old; frequency, external causes and outcome of potentially injurious BW incidents; and environmental risk factors such as number of floor levels, stairs, and swimming pools. Incidents mainly involve external causes of hitting objects, flipping over on flat surfaces, accessing dangerous items, falling down stairs, and falling into pools. Severity was categorized as a potentially injurious incident, emergency room (ER) visit, hospitalization, long-term disability, and death. For long-term disability, the respondent was asked to specify it, i.e., provide details. Clearly where the patient was not taken for clinical assessment or even if they were, the sibling would not be generally be expected to provide a diagnosis, only the external cause. For falls down stairs, it would be surprising if there were no injury at all as such incidents tend to be severe. The questionnaire was improved after a pilot among grade 12 students in a randomly selected class. It was explained and distributed by the Arab medical student investigators and completed in class.

### Data processing and analysis

Questionnaires were pre-coded for data entry, verified after completion, double- entered and compared to prevent keystroke, range, and consistency errors, and then transferred to SPSS. Analysis included regrouping, frequencies, and cross-tabulations. For incidence density calculation, average exposure per baby was assumed to be 6 months, based upon an Irish study where median duration of use was 26 weeks, starting at 26 and finishing at 54 weeks (Garrett et al 2002). In Canada, range of use was estimated at 5–14 months by Health Canada (Health Canada 2007). Confidence intervals were calculated using the method recommended by Newcombe and Altman (2000). For comparisons of injury rate ratios between families living on one versus more than one level, odds ratios were computed; since more national families lived in multi-level homes, these data were also stratified into national and non-national families to assess potential confounding by nationality.

## Results

### Incidents, injuries, and external causes

A total of 696 students completed the survey, 55 % (*n* = 385) of whom were Emirati citizens. Response was 100 %. Prevalence of BWs in families was 87 % (*n* = 605/696), with a total of 2376 children exposed to BW use (Table 1). In 49 % of families (*n* = 297/605) where a BW had been used, at least one child was reported injured, with a total of 646 injuries (Table 2); 52 % (*n* = 180) of victims were Emiratis and 42 % (*n* = 117) other Arabs. Of the 646 injuries, 118 were treated in emergency rooms (ER), 42 hospitalized, 11 long-term disabled, and 3 died (Table 2). Although the nature of most injuries was unknown, 23 ER visits were for head injury, 12 for lacerations, 3 for fractures, and 3 for drug ingestion or other poisoning. One infant in a BW was killed by a car, while external causes of the 2 other deaths were unspecified.

**Table 1:** Number of users of baby walkers and of injured babies per family and total number of injuries, families of female Grade-12 students, Al Ain, United Arab Emirates, 2005 (*n* = 696 families; 2466 children)

No. of children who used BW per family	No. of families	%	No. of children who used a BW	No. of BW injured babies per family	Injured babies		Injuries
					No.	%	No.^d^
1	104	15	104	1	144	23	144
2	122	18	244	2	71	11	142
3	91	13	273	3	33	5	99
4	85	12	340	4	22	4	88
5	69	10	345	5	11	2	55
>5	134	19	1070^a^	>5	16	3	118^a^
Total used BW	605	87	2376		297	48	646
None	90	13	90	None	330	53	
Total	695^b^	100	2466	Total	627	101^c^	

Note: ^a^Multiple categories involved to give total; ^b^1 missing value;^c^ percent does not total 100 due to rounding; ^d^ > one injury per child sometimes occurred

**Table 2:** Number and rate of events related to baby walker use, families of female Grade-12 students, Al Ain, United Arab Emirates, 2005 (*n* = 2376 BW users in 695 families)

BW-related events		Incidence density of babywalker-related events			
Type	Number	No. per 1000 babies	No. per 1000 baby years	95 % CI	Ratio of events per “x” no. of BW users
Incidents	2759	2322	1161	(1144,1179)	1 per user
Injuries	648	544	272	(254,290)	1 per 4 users
ER^a^ visits	118	100	50	(41,58)	1 per 20 users
Hospitalizations	42	36	18	(12,23)	1 per 55 users
Disabilities	11	10	5	(2,7)	1 per 200 users
Deaths	3	2.6	1.3	(1.2,2.9)	1 per 1000 users

Note: ^a^ *ER*, emergency room

With 2376 children in the students’ families exposed to use of a BW for an estimated average of six months, it was possible to estimate incidence densities for 1188 baby-years of exposure (Table 2). BW-related events could then be expressed per 1000 baby-years (double the rate per 1000 babies), as well as the anticipated probability of each event, ranging from a risk of 1 potentially injurious incident per user, to 1 long-term disability per 200 users and 1 death per 1000 users.

The most frequent external cause of a potentially injurious BW incident was hitting a hard object, accounting for nearly half of all 2759 incidents (Table 3). Next were flipping over, accessing dangerous items, and falling down stairs. The potentially most dangerous incidents included 300 falls down stairs and 32 into swimming pools. Stair-related injuries were more frequent in two-story homes while in one-story homes, overturning on flat surfaces and accessing dangerous items.

**Table 3:** Number of baby walker incidents by external cause, number of floors in the home, and nationality, families of female Grade-12 students, Al Ain, United Arab Emirates, 2005 (*n* = 696)^a^

External cause of injury			Number of stories in home				Nationality			
	Total		1 (*n* = 440)		≥2 (*n* = 253)		UAE (*n* = 385)		Other (*n* = 311)	
	n	%	n	%	n	%	n	%	n	%
Hitting hard object^b^	1322	48	783	47	537	49	685	49	637	47
Flipping over on flat surface	632	23	410	25	222	20	301	21	331	25
Accessing dangerous item^c^	473	17	309	18	163	15	202	14	271	20
Falling down stairs	300	11	143	9	157	14	200	14	100	7
Falling into swimming pool	32	1	18	1	14	1	21	2	11	1
Total	2759	100	1663	100	1093	99^d^	1409	100	1350	100

^a^ Number of incidents exceeds number of families; ^b^ includes furniture; ^c^ includes knives, poisons, electrical outlets, ^d^ percent does not total 100 due to rounding

### Home environmental determinants for BW injury

84 % (*n* = 573) of students’ families lived in houses and 16 % (*n* = 109) in apartments. 64 % (*n* = 440) of houses and apartments had one story and 36 % (*n* = 253) ≥2; 48 % (*n* = 186) of UAE nationals and 22 % (*n* = 67) of non-nationals lived in a residence with ≥2storys. External stairways are generally much lower than internal; however, falls occurred on both. 10 % (*n* = 69) of residences had a sunken room and 21 % (*n* = 142) one or two steps between or within rooms, while 7 % (*n* = 45) had an elevated room. 82 % (*n* = 517) of families used BWs downstairs inside the home, 15 % (*n* = 92) upstairs and downstairs, and 3 % (*n* = 21) only upstairs. 36 % (*n* = 235) used a BW outside in yards, 6 % (*n* = 38) on verandas, and 4 % (*n* = 26) in driveways.

BW hospitalizations were more than double in frequency among families in residences with ≥2 storeys compared with one, while long-term disabilities were 17 times greater (Table 4). After stratification of UAE nationals and non-nationals, results were similar for all categories except hospitalizations, where odds ratios were non-significant for nationals, but 14.6 (95 % CI 4.7, 45.7) for non-nationals.

**Table 4:** Number and odds ratio of events related to baby walker use by number of stories in home, families of female Grade-12 students, Al Ain, United Arab Emirates, 2005 (*n* = 2376 BW users in 605 families)

BW-related events	Number of stories in home				Odds ratio	95 % CI
	1		>1			
	Number (yes/yes + no)	%	Number (yes/yes + no)	%		
Fell down stairs	143/466	31	157/325	48	2.1	1.6, 2.8
Injured	260/472	55	386/501	77	2.7	2.1,3.6
ER^a^ visit	68/421	16	50/252	20	1.3	0.9, 1.9
Hospitalization	18/399	5	24/248	10	2.3	1.2, 4.3
Disability	1/392	.3	10/242	4	16.8	2.1, 132.5

Note: ^a^ *ER*, emergency room

12 % (*n* = 83) of families had a home swimming pool. Only 12 % (*n* = 10) of pools had complete automatic passive protection by a self-closing and self-latching gate, while 60 % (*n* = 49) had no gate, 21 % (*n* = 17) manually closing gates, 6 % (*n* = 5) self-closing, and 1 % (*n* = 1) other.

## Discussion

### Incidence

Despite potential limitations of lifetime recall for students, severe and minor non-fatal BW injuries as well as deaths were frequent. In nearly half of UAE families using BWs, at least one child sustained injury, averaging two injuries per family. There were 50 ER visits, 18 hospitalizations, 5 disabilities and 1 death per 1000 infant years. As a comparison, in the United States, annual incidence of BW emergency department visits was estimated at 8.9 injuries per 1000 infants, severe 1.7 per 1000 (Chiaviello et al. 1994). During 1973–1998, 34 BW-related deaths were reported (American Academy of Pediatrics 2001). With an average population of about 4 million infants (births) at risk over 25 years, i.e., 100 million infant years, US data at 0.034 BW deaths per 100,000 infant (births) years may be incomplete or the risk far less, since our UAE study found 3 deaths in a city of less than 0.5 million, about 9000 infants (births) per year with a recall period of perhaps as much as 18 years, say about 1.9 deaths per 100,000 infant years. The ratio would be about 55 to 1 for UAE to USA. These are approximate estimates based on small UAE numbers, but provide some possibilities as to differences in BW mortality risk for infants in the two countries. For two of the UAE BW deaths, the external cause was not specified by the sibling, possibly because the term “specify” was unclear in translation or because the sibling was crying at that point, as all three were.

### Built environment factors

Homes are the main site for severe BW injuries (Ozanne-Smith and Brumen 1993; Thein et al. 2005; Shields and Smith 2006). Although hitting a hard object and flipping over on a flat surface were the most frequent BW injury incidents in the UAE, most dangerous were falls down stairs and into pools. BW injuries were frequent due to the combination of high prevalence of BWs and of home stairs. Many families, especially citizens, live in multi-story residences with inside and outside stairs. For multi-story residences, risk of BW hospitalization was more than doubled and long-term disability was 17 times greater. Other frequent hazards include more than one floor level on the same story, in-ground pools, and play areas contiguous with parking and/or traffic. Most floors and outside play areas are hard finished surfaces (Al-Saridi et al. 2005). Basements are uncommon in desert homes in the UAE, whereas in the United States about 40 % of BW injuries occur on basement stairs (Consumer Product Safety Commission as cited in American Academy of Pediatrics 2001).

In Australia, 65 % of households using BWs had stairs (Ozanne-Smith and Brumen 1993). 77 % of BW injuries involved a fall, and stair falls accounted for 47 % of BW hospitalizations. This and our UAE results contrast with the city of Baghdad, where most families reportedly lived in single-story homes and only 2 of 148 injuries involved stairs (Al-Nouri and Al-Isami 2006). In the US, 96 % of BW injuries treated in ERs were stair falls (Smith et al. 1997). Among BW injuries at a trauma centre, 95 % were stair falls (Partington et al. 1991). In Australia, only 30 % of households had protection such as gates at top and bottom of stairs; impact with concrete or other fabricated surfaces was the direct cause of injury in 50 % of BW incidents (Ozanne-Smith and Brumen 1993). Unfortunately, not all stair gates adequately prevent BW injuries (Smith et al. 1997).

Home pools are another hazardous built environment, and in our study 31 incidents involved infants in BWs falling into pools. Exposure is high, with 12 % of families having pools in this study. In other unpublished population-based studies in five UAE cities, 9 to 29 % of families had home pools, fewer than 10 % with automatic self-closing and self-latching gates (Al-Saridi et al. 2005; Mussab et al. 2006). Although other medical students described at least one BW family pool death, in our study infants survived pool incidents, suggesting presence of a caregiver(s). In a US report of 11 BW deaths, 4 were drownings, at least 3 in pools (US Consumer Product Safety Commission 1994).

Although little is published on BW injuries in yard play areas contiguous with parking, one death in our study resulted from a vehicle. Most homes with yard play areas have smooth surfaces continuous with parking areas, often opening into a street. Hence, risk for infants in rapidly moving BWs is potentially high. In New Zealand fewer child injuries by family vehicles occurred with barriers between home play areas and parking (Roberts et al. 1995).

### Anatomical site of injury

Among UAE children with BW injuries taken to ER, the head was the most frequent anatomical location of injury, 20 % of the total including unknowns. Level of energy transfer to a child’s body in falls is determined by distance fallen and energy-absorbing capacity of the impacted surface (Smith et al. 1997). The number of steps fallen in a BW is associated with hospital admission and head injury such as skull fracture (Smith et al. 1997; Al-Nouri and Al-Isami 2006). While many injuries are minor, concussion, intracranial haemorrhage, and fractures of the skull and cervical spine do occur with stair falls (Al-Nouri and Al-Isami 2006; Health Canada 2007; American Academy of Pediatrics 2001; Taylor 2002). Elsewhere, in Iraq 94 % of 83 children using BWs sustained one or more injuries (*n* = 148) between 6–10 months of age; with 82 % involving the head (Al-Nouri and Al-Isami 2006). Among US emergency room BW injuries, 91 % involved the head and 62 % of fractures the skull (Shields and Smith 2006; Casell et al. 1997). In Sweden, BWs were the childcare product most frequently associated with concussion or “mild” traumatic brain injury for the entire 0–4-year-old population in 1998–99 (Emanuelson 2003).

### Limitations of study population

Although research was confined to final year classes, duplicate reporting by siblings or other relatives could have occurred, and sampling was cluster-based at the level of class. However, student respondents provided the optimal possibility for a population-based sample representative of all prospective future Arab mothers in the study city. Furthermore, once students leave home for marriage, work, or advanced study, their exposure to and awareness of infant injuries in the family home would decline. In non-school populations, 100 % response is unusual; however, 98–100 % response is frequent in our medical student school surveys. High school students are enthused to meet medical students and enjoy a break from school routines.

Many Emirati and other Arab mothers are nowadays highly educated and work, with childcare provided by expatriate housemaids. Older siblings are often delegated by parents to keep an eye on younger children and care by housemaids. Siblings can be more aware of incidents than parents since paid caregivers may fear reporting incidents. In local Islamic cultural contexts, home surveys of women are challenging and it can also be difficult to obtain non-biased samples of mothers in other settings. Finally, large sample sizes are necessary to detect sufficient uncommon fatal or disabling incidents, which would not be feasible if studies involved interviewing individual mothers, some with only a single child.

Population surveys are useful for estimating injury frequency. Victims or their families are allowed to speak for themselves when the health system is unable to do so (Fingerhut and McLoughlin 2001). For BWs, lack of a specific ICD-10 code for such product-related injuries complicates assessment of burden of injury. BW injuries may be counted under falls, motor vehicle injuries, immersions, or other external cause codes (World Health Organization 2007a).

### Limitations of recall period

Our grade-12 potential rather than actual mothers had long recall periods for all BW injuries during their childhood and youth. Less severe incidents could have been forgotten, leading to recall bias with underreporting or misreporting of frequency, nature, severity, and external causes of non-fatal and non-disabling injuries, so-called loss of memory (Harel et al. 1994) or memory decay (Mock et al. 1999; Moshiro et al. 2005). Alternatively, recall bias can lead to reporting of incidents outside the recall period, known as telescoping; however, this seems unlikely in the current context.

Loss of memory reportedly is minimal for “severe injuries”, defined in the US as “injuries resulting in at least 1 bed day, 1 school loss day, surgery, or hospitalization” (Harel et al. 1994). The frequency per BW user of our three more severe categories, deaths, disabilities, and hospitalizations, should be if anything conservative if undercounting occurred. ER visits, injuries, and incidents could be expected to be undercounted, with study data representing lower limits in a sensitivity analysis based on scaled recall assumptions. This could explain ER visit and death ratios between UAE and the USA of 5 and 55 respectively. Reporting practices and use of ER versus clinics and definitions of severity can differ among countries. In Greece, to classify injuries as “major” required only contact with a health institution (Petridou et al. 2004).

Although reports validating recall periods greater than a year for injury are uncommon, fatal injury in a BW would not generally be forgotten in a lifetime, and indeed three students with loss of a sibling began crying during the questionnaire. A child with permanent disability would be a daily reminder of BW injury even decades before. Elsewhere, reported trauma deaths were highly accurate (Claude et al. 1984; Snow et al. 1992; Snow et al. 1993), and recall by relatives of a family member’s death was unaffected by recall period (Claude et al. 1984). In evaluating maternal recall of child deaths from all causes in Bangladesh, no difference was found between one and five years post-incident (Halder et al. 2009).

### Other potential limitations

Since some families move, actual built environments for some BW injuries may have differed from reported. However, families do not relocate frequently as in some countries and due to government subsidies many citizens build homes in their community shortly after marriage. Some are assigned a house or apartment elsewhere by employers but return home to families on weekends. For expatriates, it is not easy to relocate or change employers or housing due to work permit requirements, rules on switching employers, and the fact that houses are generally assigned for the duration of work contracts. Nonetheless, research was conducted in one of four main UAE cities and the situation could differ elsewhere. Abu Dhabi and Dubai have more high-rise apartments and possibly less exposure to stairs, pools, and play areas contiguous with parking, than Al Ain, where most buildings greater than four storeys have been prohibited for aesthetics.

Our incidence data were based upon an estimated exposure denominator of six months per baby, similar to research elsewhere. However, if exposure were doubled to 12 months per baby, unlikely considering when an average baby stands and walks, then sensitivity analysis would halve risk to 1 death per 2000 users, 1 disability per 400, 1 hospitalization per 110, 1 ER visit per 40, and 1 injury per 8.

Another exposure issue is the number of hours per day infants are left in a walker. There could be major differences among families and even among siblings and we are not aware of studies documenting this and our survey could not have readily addressed it.

With no published data on ER visits, hospitalizations, and deaths from BWs in the UAE, validation of student recall was challenging. However, data from a subsequent intervention for BW injuries among new mothers support injury frequency and severity in this study and indirectly students as rapporteurs of BW injury of siblings. Among 339 respondents, pre-intervention data for as far back as could be recalled found 259 children injured, 57 taken to ER, 13 hospitalized, 15 left disabled, and 0 deaths. During a single post-intervention year, 110 available respondents reported 9 children treated for BW injury at primary health care centres, 17 in ER, 3 hospitalizations, 1 disability, and 2 deaths. This suggests that in environments with difficult access, interview, and prospective follow-up of sufficient mothers, interview of high-school girls using long recall periods is a valid option to estimate incidence of severe BW injuries.

## Conclusion

BW injuries are serious contributors to UAE infant mortality and morbidity. High incidence and severity are due to high prevalence of BWs, built environment hazards, and other factors. These are a result of a lack of product safety regulations to ban BWs. Other cities in the UAE and countries elsewhere also merit study of variables such as prevalence of BWs, exposure to stairs and differences in floor levels, as well as automatically closing and latching child barriers between play areas and pools, family parking, and streets.

In an earlier paper associated with this study (Grivna et al. 2015), we reported on why families used BWs and perceptions regarding causes of injuries. 84 % reported that BWs are used to keep babies safe and 92 % to help them walk earlier. 70 % attributed BW injuries to carelessness of parents, 43 % carelessness of baby, 42 % fate or destiny, 14 % evil eye, and 3 % jinns. In contrast, only 16 % perceived the BW and 40 % dangerous environments as causal.

Clearly, in the short term, active protection by health promotion is warranted to correct misperceptions about safety and efficacy of BWs and causality of incidents and injuries. Until countries have been cleared of BWs, families need frequent warnings to avoid and dispose of them (Emanuelson 2003). Concurrently, automatically closing and latching childproof barriers should be mandatory to separate play areas from stairs, pools, and vehicles, and even helmets might be considered if stairs are easily accessible in a home. However, since such measures require repeated intervention at household and sales levels, none are adequate substitutes for legislation blocking import and sales. Supervision by parents, older siblings or others cannot be relied upon, since an infant in a BW can move at 90cm/s (3ft/s), so there is often not time to react (American Academy of Pediatrics 2001). Supporting this are reports that many incidents occur with parents in the room (American Academy of Pediatrics 2001; US Consumer Product Safety Commission 1994; Millar et al. 1975).

Since a substantial proportion of BW injuries result from access to hazards other than stairways, stationary play centres are considered safer, more comprehensive, and more practical alternatives for caregivers than mobile BWs designed not to roll down a stairway (Thompson 2002). Modified mobile devices too large to pass through a standard door and with brakes were a compromise solution adopted in the USA, and provide incomplete protection compared to banning all BWs. It is a voluntary standard and compliance has been limited (American Academy of Pediatrics 2001). Furthermore, such devices do not change the negative impact of BWs on physical development of infants, especially the risks for infants with neuromuscular disorders. Infants in mobile BWs can still reach dangerous items such as poisons, hot objects, and knives, while burns have been frequent in some studies (Ozanne-Smith and Brumen 1993; Liao and Rossignol 2000; Martin 2003).

To provide long lasting passive or automatic protection, governments worldwide, including the Middle East, should review the expert testimony and evidence given to the Canadian government’s Board of Review and results of on-going surveillance (Public Health Agency of Canada 2009), and prohibit BW imports and sales. An initial voluntary industry ban was effective in Canada while regulations were implemented. A ban on manufacture and sales is supported by the American Academy of Pediatrics (2001). In the UAE, if there are no manufacturers, then a ban could be focused mainly on importation and sales. While diligent enforcement is essential, this is required for all hazardous toys and should not excuse inaction. The BW issue deserves urgent addition to priority lists for advising governments on injury interventions (World Health Organization 2007b) of the Division of Injuries and Violence at the World Health Organisation and for the agenda of the European Commission’s Directorate General for “Health and Consumers”.

Lobbying by relevant national groups can be key to encouraging governments to act. In Canada, the Canadian Paediatric Society’s Injury Prevention Committee led a long term evidence-based campaign. In the UAE, paediatric and other medical and health societies could identify and collaborate with various safety oriented groups such as health authorities, Red Crescent, and other organizations.

## References

[CR1] Al-Baladi AMY. Care of pregnant women, infants and children. Haj Kassem M. Baghdad Ministry of Culture and Information, 1980; 207. Cited in: Al-Nouri L, Al-Isami S. Baby walker injuries. Ann Trop Paediatr*.* 2006; 26:67-71. (Original Arabic textbook: Al Baladi, Ahmad bin Mohamad. Management of pregnant women, babies and children, protection of their health and treatment of their diseases. Publication date and page unknown, author deceased in 990). 10.1179/146532806X90637 16494707

[CR2] Al-Nouri L, Al-Isami S (2006). Baby walker injuries. Ann Trop Paediatr.

[CR3] Al-Saridi A, Al-Darmaki A, Anoohi R, Al-Echtibi S, Grivna M, Barss P (2005). Knowledge, attitude and practice of selected female students for unintentional child home injuries.

[CR4] American Academy of Pediatrics. Committee on Injury and Poison Prevention (2001). Injuries associated with infant walkers. Pediatrics.

[CR5] Cassell OC, Hubble M, Milling MA, Dickson WA (1997). Baby walkers--still a major cause of infant burns. Burns.

[CR6] Chiaviello CT, Christoph RA, Bond GR (1994). Infant walker-related injuries: a prospective study of severity and incidence. Pediatrics.

[CR7] Claude J, Eilber U, Chow KW, Frentzel-Beyme R (1984). Validity of cause of death statements from relatives. Int Arch Occup Environ Health.

[CR8] Emanuelson I (2003). How safe are childcare products, toys and playground equipment? a Swedish analysis of mild brain injuries at home and during leisure time 1998-1999. Inj Control Saf Promot.

[CR9] Fingerhut LA, McLoughlin E, Rivara FP, Cummings P, Koepsell TD, Grossman DC, Maier RV (2001). Classifying and counting injury. Injury control a guide to research and program evaluation.

[CR10] Garrett M, McElroy AM, Staines A (2002). Locomotor milestones and babywalkers: cross sectional study. BMJ.

[CR11] Grivna M, Barss P, Al-Hanaee A, Al-Dhahab A, Al-Kaabi F, Al-Muhairi S (2015). Baby walker injury awareness among grade-12 girls in a high-prevalence Arab country in the Middle East. Asia Pac J Public Health.

[CR12] Halder AK, Gurley ES, Naheed A, Saha SK, Brooks WA, El Arifeen S, Sazzad HM, Kenah E, Luby SP (2009). Causes of early childhood deaths in urban Dhaka, Bangladesh. PLoS One.

[CR13] Harel Y, Overpeck MD, Jones DH, Scheidt PC, Bijur PE, Trumble AC, Anderson J (1994). The effects of recall on estimating annual nonfatal injury rates for children and adolescents. Am J Public Health.

[CR14] Health Canada. Board of Review Inquiring into the Nature and Characteristics of Baby Walkers. 2007 Available at URL: http://www.marketwired.com/press-release/health-canada-canadas-new-government-accepts-recommendation-baby-walker-board-review-740656.htm. Accessed 20 Mar 2016.

[CR15] Health Canada (2002). Testing of baby walkers: test report.

[CR16] Liao CC, Rossignol AM (2000). Landmarks in burn prevention. Burns.

[CR17] Martin HC (2003). Injury caused by baby walker. Med J Aust.

[CR18] Millar R, Colville J, Hughes NC (1975). Burns to infants using walking aids. Injury.

[CR19] Mock C, Acheampong F, Adjei S, Koepsell T (1999). The effect of recall on estimation of incidence rates for injury in Ghana. Int J Epidemiol.

[CR20] Moshiro C, Heuch I, Astrøm AN, Setel P, Kvåle G (2005). Effect of recall on estimation of non-fatal injury rates: a community based study in Tanzania. Inj Prev.

[CR21] Mussab A, Nassir A, Helal I, Karami A, Barss P, Grivna M, Bernsen R (2006). Water safety and drowning prevention in the east coast cities of the United Arab Emirates.

[CR22] Newcombe RG, Altman DG. Proportions and their differences. Chapter in: Altman DG, Machin D, Bryant TN, Gardner MJ. Statistics with confidence. 2nd Ed. British Medical Association, BMJ Books; 2000:45-88.

[CR23] Ozanne-Smith J, Brumen I (1993). The safety of babywalkers. Hazard.

[CR24] Partington MD, Swanson JA, Meyer FB (1991). Head injury and the use of baby walkers: a continuing problem. Ann Emerg Med.

[CR25] Petridou E, Dessypris N, Frangakis CE, Belechri M, Mavrou A, Trichopoulus D (2004). Estimating the population burden of injuries: a comparison of household surveys and emergency department surveillance. Epidemiology.

[CR26] Public Health Agency of Canada. Child and Youth Injury in Review – Spotlight on Consumer Product Safety, 2009. Ottawa, ON, Canada at URL http://www.phac-aspc.gc.ca/publicat/cyi-bej/2009/index-eng.php. Accessed 20 Mar 2016.

[CR27] Ridenour M (1997). How effective are brakes on infant walkers?. Percept Mot Skills.

[CR28] Roberts I, Norton R, Jackson R (1995). Driveway-related child pedestrian injuries: a case-control study. Pediatrics.

[CR29] Rodgers GB, Leland EW (2005). An evaluation of the effectiveness of a baby walker safety standard to prevent stair-fall injuries. J Saf Res.

[CR30] Santos Serrano L, Paricio Talayero JM, Salom Pérez AO, Grieco Burucúa M, Martín Ruano J, Benlloch Muncharaz MJ, Llobat Estellés T, Beseler SB (1996). Patterns of use, popular beliefs and proneness to accidents of a baby walker (go-cart). bases for a health information campaign. An Esp Pediatr.

[CR31] Shields BJ, Smith GA (2006). Success in the prevention of infant walker-related injuries: an analysis of national data, 1990-2001. Pediatrics.

[CR32] Smith GA, Bowman MJ, Luria JW, Shields BJ (1997). Babywalker-related injuries continue despite warning labels and public education. Pediatrics.

[CR33] Snow RW, Armstrong JR, Forster D, Winstanley MT, Marsh VM, Newton CR, Waruiru C, Mwangi I, Winstanley PA, Marsh K (1992). Childhood deaths in Africa: uses and limitations of verbal autopsies. Lancet.

[CR34] Snow RW, Basto De Azevedo I, Forster D, Mwankuyse S, Bomu G, Kassiga G, Nyamawi C, Teuscher T, Marsh K (1993). Maternal recall of symptoms associated with childhood deaths in rural east Africa. Int J Epidemiol.

[CR35] Taylor B (2002). Babywalkers: delay development, cause injuries, and we should consider banning them. BMJ.

[CR36] Thein MM, Lee BW, Bun PY (2005). Childhood injuries in Singapore: a community nationwide study. Singapore Med J.

[CR37] Thompson PG (2002). Injury caused by baby walkers: the predicted outcomes of mandatory regulations. Med J Aust.

[CR38] US Consumer Product Safety Commission (1994). Baby walkers: advance notice of proposed rulemaking. Fed Regist.

[CR39] World Health Organization (a). Tabular List of inclusions and four-character subcategories, Chapter XX, External causes of morbidity and mortality (V01-Y98). In: International Statistical Classification of Diseases and Related Health Problems. Geneva: 10th Revision, Version for 2010. http://apps.who.int/classifications/apps/icd/icd10online/. Accessed 20 Mar 2016.

[CR40] World Health Organization (b). Preventing injuries and violence: a guide for ministries of health. Geneva: WHO; 2007:23-26.

